# News and Coming Events

**Published:** 1909-07-31

**Authors:** 


### News and Coming Events.

Mr. Sydney Scott, M.S., F.R.C.S., has been elected surgeon for diseases of the ear and throat to the National Hospital for the Relief and Cure of the Paralysed and Epileptic, vice Mr. Cumberbatch and Sir Felix Semon.

The sixth annual meeting of the Queen Alexandra Sanatorium, Davos, was held at 11 Chandos Street, W. Lord Balfour of Burleigh, who presided, said he had lately visited the sanatorium; and since the building was finished there was no reason why it should not be opened in October. The Council expect to complete the building within the original estimate of £36,000. A donor, who wished to remain anonymous, had placed the sum of £25,000 at the disposal of the Council. The first charge on that sum would be the opening of the institution free of debt, and the rest of the money would be devoted, at the discretion of the Council, to the further extension of the sanatorium, the installation of Röntgen rays apparatus, and such other purposes as the Council might deem necessary.

The annual meeting and dinner of the Brussels Medical Graduates' Association was held at the Garden Club in the "White City," Shepherd's Bush, on July 15, under the chairmanship of Dr. Richard Paramore, the president. Dr. Oliver, the guest of the evening, proposed the health of the Association, and this toast was replied to by the hon. secretary, Dr. Arthur Haydon. Dr. Haydon announced with satisfaction that, following his interview last year at Brussels with the University authorities, every one of the recommendations made by the Council has been accepted and carried into official effect. Thus at last the Association has been officially recognised by the University of Brussels. Dr. R. Paramore, Dr. Major Greenwood, and Dr. Arthur Haydon were elected respectively, president, hon. treasurer, and hon. secretary for the ensuing year.

The latest British Pharmaceutical Conference was opened at Newcastle-on-Tyne on Monday evening, July 26, at the Armstrong College. The Lord Mayor and Professor Lebour, vice-principal of Armstrong College, delivered addresses of welcome. In the lecture theatre demonstrations were given, and various experiments were publicly conducted in the Armstrong Laboratory, the Herschel Laboratory, and in the electrical engineering department.

Col. Needham presided on July 26 at the half-yearly court of governors of the East London Hospital for Children. During the past six months in-patients numbered 958. The number of new out-patients was 16,344, and the attendances numbered 45,521. The expenditure for the half-year was £5,800. The excellent financial condition reported at the close of last year has unfortunately not been maintained, and there is now a deficit of £1,100. Plans are in preparation for constructing and equipping a much needed out-patient operating theatre, and for providing better accommodation for the casualty officers.

At the closing session of the Health Congress at Leeds, Dr. D. A. Carruthers read a paper on "The Aims of Medical Examination." Dr. Kaye advocated early treatment of defects in children, and several papers were read upon "The Control of Infectious Diseases," while Dr. Thos. Orr contributed a paper on the mode of dissemination of diphtheria in schools. A resolution urging that, whether the Milk and Dairies Bill were passed or not this session, the Tuberculosis Order should not be withdrawn, was carried by a bare majority, and was subsequently withdrawn. Of the earlier papers read during last week, that on Preventive Medicine by Dr. Arthur Newsholme attracted especial notice.

